# Retraction: The anchoring of a Cu(ii)–salophen complex on magnetic mesoporous cellulose nanofibers: green synthesis and an investigation of its catalytic role in tetrazole reactions through a facile one-pot route

**DOI:** 10.1039/d3ra90132g

**Published:** 2024-01-05

**Authors:** Pouya Ghamari kargar, Ghodsieh Bagherzade

**Affiliations:** a Department of Chemistry, Faculty of Sciences, University of Birjand Birjand Iran P.ghamari71@gmail.com; b Department of Chemistry, Faculty of Sciences, University of Birjand Birjand Iran gbagherzade@gmail.com bagherzade@birjand.ac.ir +98 56 32345192 +98 56 32345192

## Abstract

Retraction of ‘The anchoring of a Cu(ii)–salophen complex on magnetic mesoporous cellulose nanofibers: green synthesis and an investigation of its catalytic role in tetrazole reactions through a facile one-pot route’ by Pouya Ghamari kargar *et al.*, *RSC Adv.*, 2021, **11**, 19203–19220, https://doi.org/10.1039/D1RA01913A.

The Royal Society of Chemistry hereby wholly retracts this *RSC Advances* article due to concerns with the reliability of the data.

The XRD patterns for CuO and Fe_3_O_4_ in Fig. 3 and the EDX data in Fig. 4a and c contain repeating sections. The authors provided the raw XRD data for CuO and Fe_3_O_4_ in Fig. 3 and the raw EDX data for Fig. 4, but these were found to contain duplicated sections of data across different datasets representing different samples within this article and other articles.

The raw XRD data provided by the authors for Fe_3_O_4_ in Fig. 3 of this article was identical in a number of different regions to the raw XRD data provided by the authors for CuO in Fig. 3 of this article and Fig. 4b of ref. 1.

The raw EDX data for Fe_3_O_4_ in Fig. 4a of this article was found to have duplicated sections of data within the raw dataset and with the raw data provided by the authors for (Fe_3_O_4_@NFC@NSalophCu)CO_2_H in Fig. 4c of this article.

The authors have stated that they outsourced the XRD and EDX data collection to an external company.

Given the significance of these concerns, the findings presented in this paper are no longer reliable.

The authors were informed about the retraction of the article. Pouya Ghamari kargar and Ghodsieh Bagherzade have not agreed with the decision.

Signed: Laura Fisher, Executive Editor, *RSC Advances*

Date: 13^th^ December 2023

## Supplementary Material
